# Cerebral venous thrombosis in infant with pertussis: A rare complication in a hyperleukocytosis and thrombocytosis Storm, A threshold worth defining

**DOI:** 10.1016/j.htct.2026.106511

**Published:** 2026-09-04

**Authors:** Hanaa Al Alawyat, Zainab Alnazer, Abdulwahab Omara, Zein Askar

**Affiliations:** a Department of pediatrics, Division of Pediatric critical care, King Fahad Specialist Hospital - Dammam, Saudi Arabia; b Pharmacy service department, King Fahad Specialist Hospital - Dammam, Saudi Arabia

## Introduction

Pertussis, commonly known as whooping cough, is a highly contagious bacterial respiratory infection caused by the Gram-negative bacterium *Bordetella pertussis* [1,2]. Despite extensive vaccination efforts, pertussis remains a significant global health concern. According to the World Health Organization, there were 163,388 reported cases worldwide in 2023, with an estimated vaccination coverage of 84% with the diphtheria-tetanus-pertussis (DTP3) vaccine. Notably, up to 1090 cases were reported in Saudi Arabia in 2024 [3]. Globally, pertussis contributes to approximately 200,000 to 400,000 deaths annually, with 90% of these fatalities occurring in unvaccinated infants under one year of age [4].

The pathogenesis of pertussis involves a two-phase disease process characterized by initial respiratory colonization (catarrhal stage), followed by systemic effects mediated by toxins (paroxysmal stage) [1,2]. Critically ill patients often present during the paroxysmal stage, exhibiting symptoms such as a non-febrile cough, tachycardia, apnea, cyanosis, and respiratory distress [5,6]. Pertussis-associated leukocytosis, primarily driven by pertussis toxin, peaks during this stage and has been identified as an independent predictor of mortality [7,8]. The risk of mortality correlates positively with the level of leukocytosis and is frequently linked to complications, including respiratory failure, cardiogenic shock, pulmonary hypertension, and multiple organ dysfunction [9, 10, 11, 12].

Previous studies have identified several risk factors associated with fatal pertussis, including being under six months of age, pneumonia, hyperleukocytosis, lymphocytosis, and neutrophilia, as well as elevated inflammatory markers, such as C-reactive protein (CRP) [13, 14, 15, 16]. In addition to the above-mentioned risk factors, a recent meta-analysis and cohort validation study further highlighted other mortality predictors, including being under 30 days old, tachycardia exceeding 200 beats per minute, seizures, pulmonary hypertension, and a white blood cell (WBC) count >30 × 10^9^/L [17]. Early risk stratification is crucial for identifying patients who may benefit from aggressive interventions, such as leukoreduction therapies, including exchange transfusions or leukapheresis. These therapies aim to reduce leukocyte aggregates in the pulmonary and systemic microvasculature, thereby improving survival rates in critically ill infants [18,19]. Pertussis toxin has also been shown to promote platelet activation and aggregation through an interaction with platelet glycoprotein Ib, increasing intracellular calcium levels and potentially increasing the risk of thrombosis independent of leukocytosis [20]. Many reports have shown an association between high platelet count of up to 900×10^9^/L and pertussis, as highlighted by Agrawal et al. in a systematic review [21,22].

While vaccination has significantly reduced the morbidity associated with pertussis, severe complications, including pulmonary and central nervous system (CNS) manifestations, continue to occur, even in immunized infants. In the United States, reported rates of pneumonia, seizures, and encephalitis/encephalopathy among culture-confirmed pertussis cases are 14.6%, 2.2%, and 0.7%, respectively [23]. Other cerebral complications associated with pertussis are varied and may include different types of paralysis, sensory deficits, aphasia, and cognitive impairments. Pathological findings have reported issues such as meningeal and cerebral hemorrhages, meningitis, encephalitis, and thrombosis in cerebral veins and sinuses [24]. Although the pathophysiology of CNS complications is not fully understood, some researchers suggest that it may result from the direct neurotoxic effects of pertussis toxin, as well as secondary effects of hypoxia, vascular occlusions, hemorrhagic events, or latent viral infections [2]. However, mortality rates can increase dramatically (by up to 70%) when pertussis is complicated by convulsions, particularly during the peak paroxysmal phase [24]. Antibiotic therapy with macrolides is the primary treatment. It is most effective when initiated during the catarrhal phase, as this helps reduce the severity and duration of the disease and the risk of transmission [4]. Supportive care is essential, and managing hyperleukocytosis through leukapheresis or exchange transfusion can be life-saving, as these procedures aid in leukoreduction and may also eliminate circulating pertussis toxin [25]. In severe cases that are resistant to standard treatments, extracorporeal membrane oxygenation may be used; however, the outcomes in such cases are generally poor [4].

This case report describes a rare presentation of cerebral venous sinus thrombosis (CVST) in an unvaccinated infant with pertussis, hyperleukocytosis, **thrombocytosis,** and seizures. While existing literature discusses various neurological complications associated with pertussis, none addresses the presentation of CVST in children, its potential pathophysiology, or its correlation with hyperleukocytosis and **thrombocytosis**. This case report highlights the necessity of early neuroimaging in pertussis cases presenting with seizures, emphasizes the importance of maintaining a high index of suspicion for CVST, and suggests future research to establish critical leukocyte and platelet thresholds that may help facilitate timely leukoreduction, platelet reduction, and antithrombotic treatment to prevent severe neurological complications.

## Case presentation

**Figure 1:**
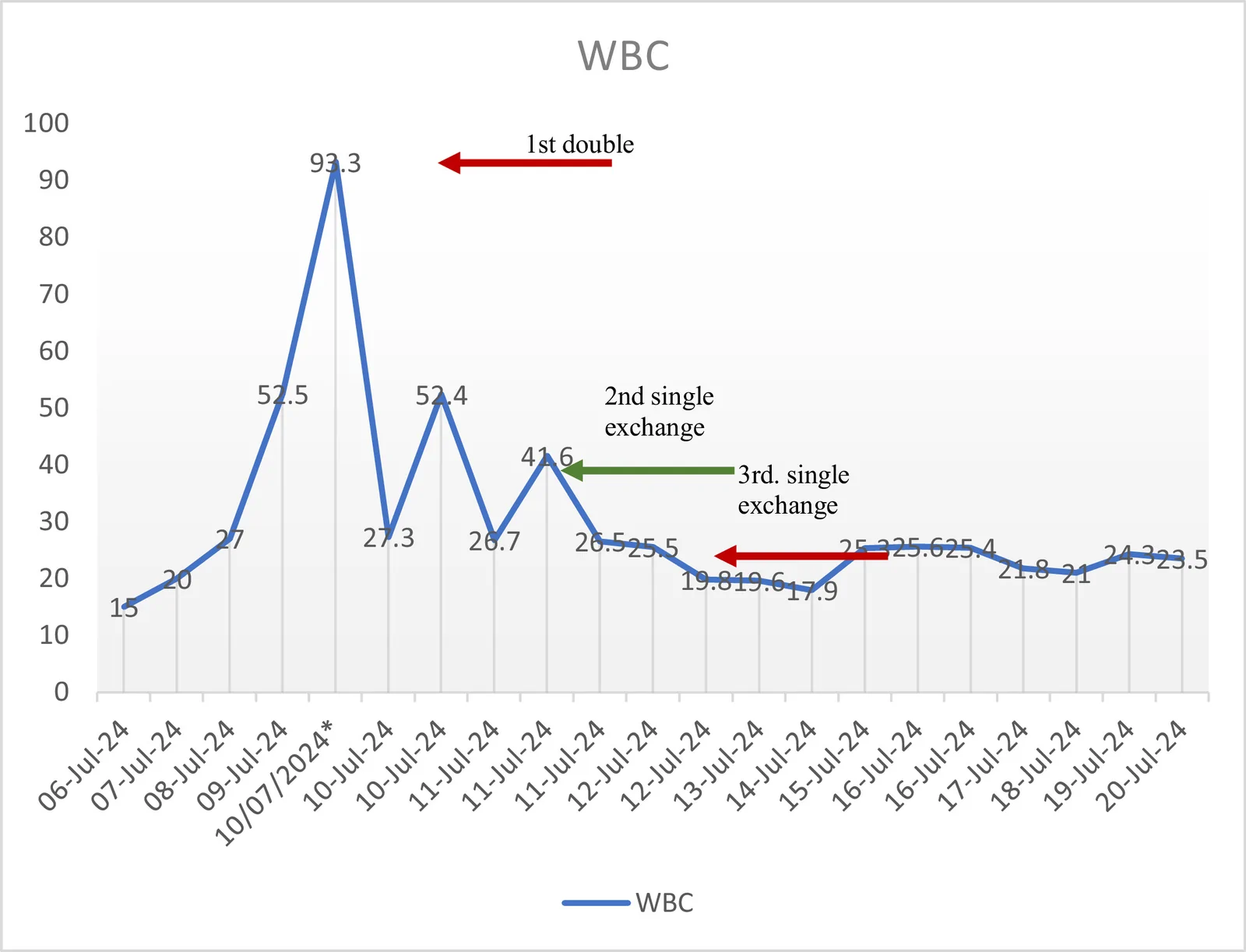
Leukocyte count by date and the timing of exchange transfusion.

A 10-week-old male infant was referred to the pediatric intensive care unit (PICU) at King Fahad Specialist Hospital in Dammam in July 2024 as a case of *Bordetella pertussis* infection complicated by hyperleukocytosis, thrombocytosis, and CVST. The patient was born full-term via an uneventful delivery, with an appropriate birth weight of 3.2 kg. His perinatal and neonatal periods were unremarkable. He had been thriving well before the current illness. His immunization history was incomplete; he had received only the birth dose of the hepatitis B vaccine and no doses of the pertussis-containing vaccine at two months of age. The infant had a brief history of close contact with siblings exhibiting upper respiratory tract symptoms. He subsequently developed a persistent cough, progressive respiratory distress, and subjective fever, with no apnea or cyanosis. He was initially evaluated at a primary care clinic on multiple occasions before being hospitalized in a private PICU for six days. A respiratory polymerase chain reaction test confirmed an infection with *B. pertussis*. He was treated with antibiotics and supported with high-flow nasal cannula oxygen. Daily leukocyte counts ranged from 15–27 × 10^9^/L. Unfortunately, on the sixth day of hospitalization, the patient experienced a generalized seizure lasting two minutes, which was aborted with benzodiazepine. At that time his WBC count had increased from 27 × 10^9^/L to 52 × 10^9^/L (Figure 1). The same day, he was transferred to our tertiary care facility for leukoreduction therapy.

**Figure 2:**
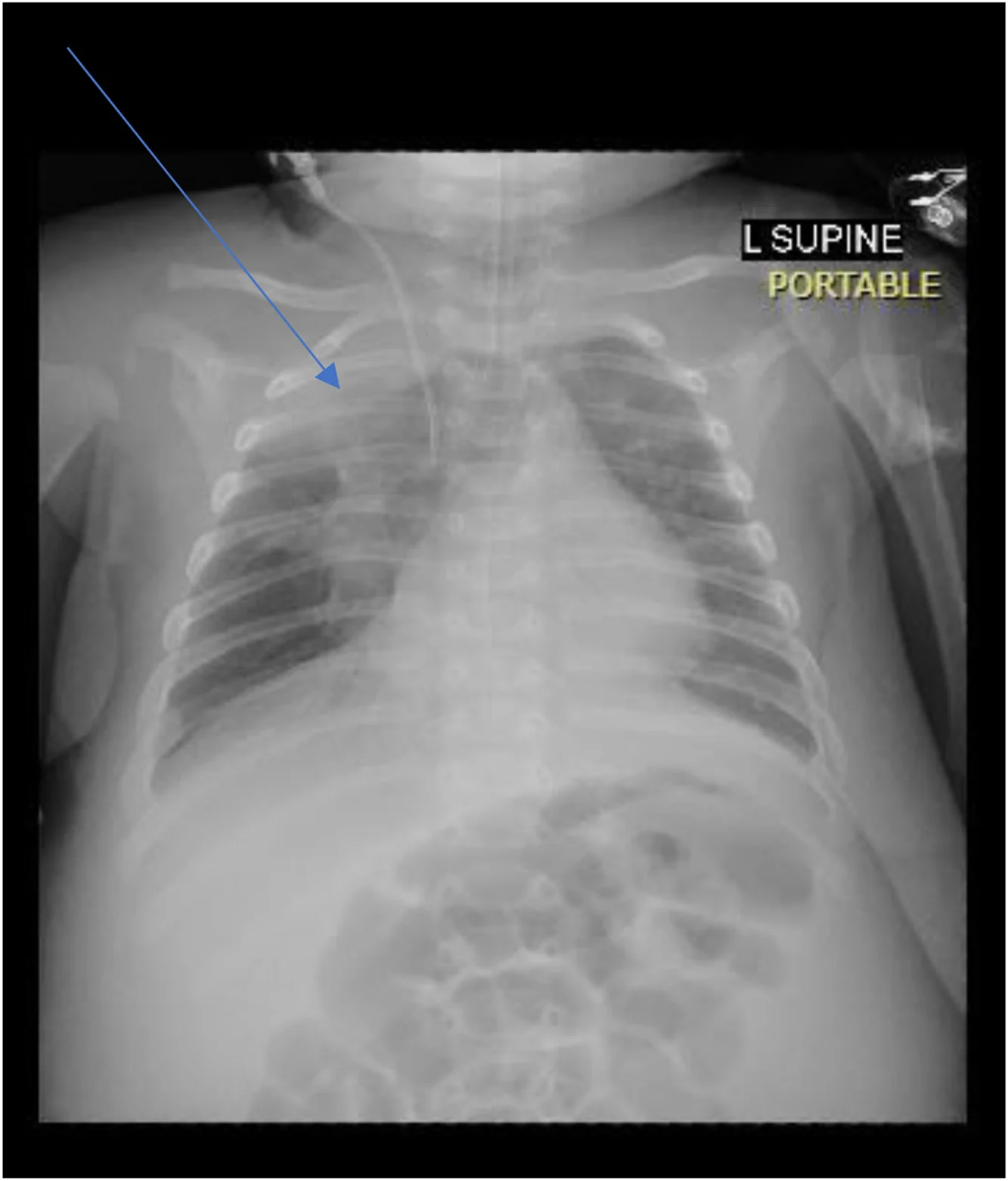
Chest radiograph showing the right sided infiltrate indicative of pneumonia (arrow).

Upon arrival, the infant was afebrile but in moderate-to-severe respiratory distress. Vital signs were notable for tachypnea (respiratory rate: 50–70 breaths/min) and tachycardia (heart rate: 150–170 beats/min), accompanied by subcostal retractions and nasal flaring. Initial laboratory investigations revealed profound leukocytosis and thrombocytosis: WBC 93.3 × 10⁹/L, absolute neutrophil count (ANC) 52.5 × 10⁹/L, absolute lymphocyte count 18.8 × 10⁹/L, and a platelet count of 1109 × 10⁹/L. Chest radiography demonstrated right upper lobe collapse along with bibasilar medial and left apical atelectasis (Figure 2). A repeat respiratory polymerase chain reaction confirmed *B. pertussis* infection.

**Figure 3:**
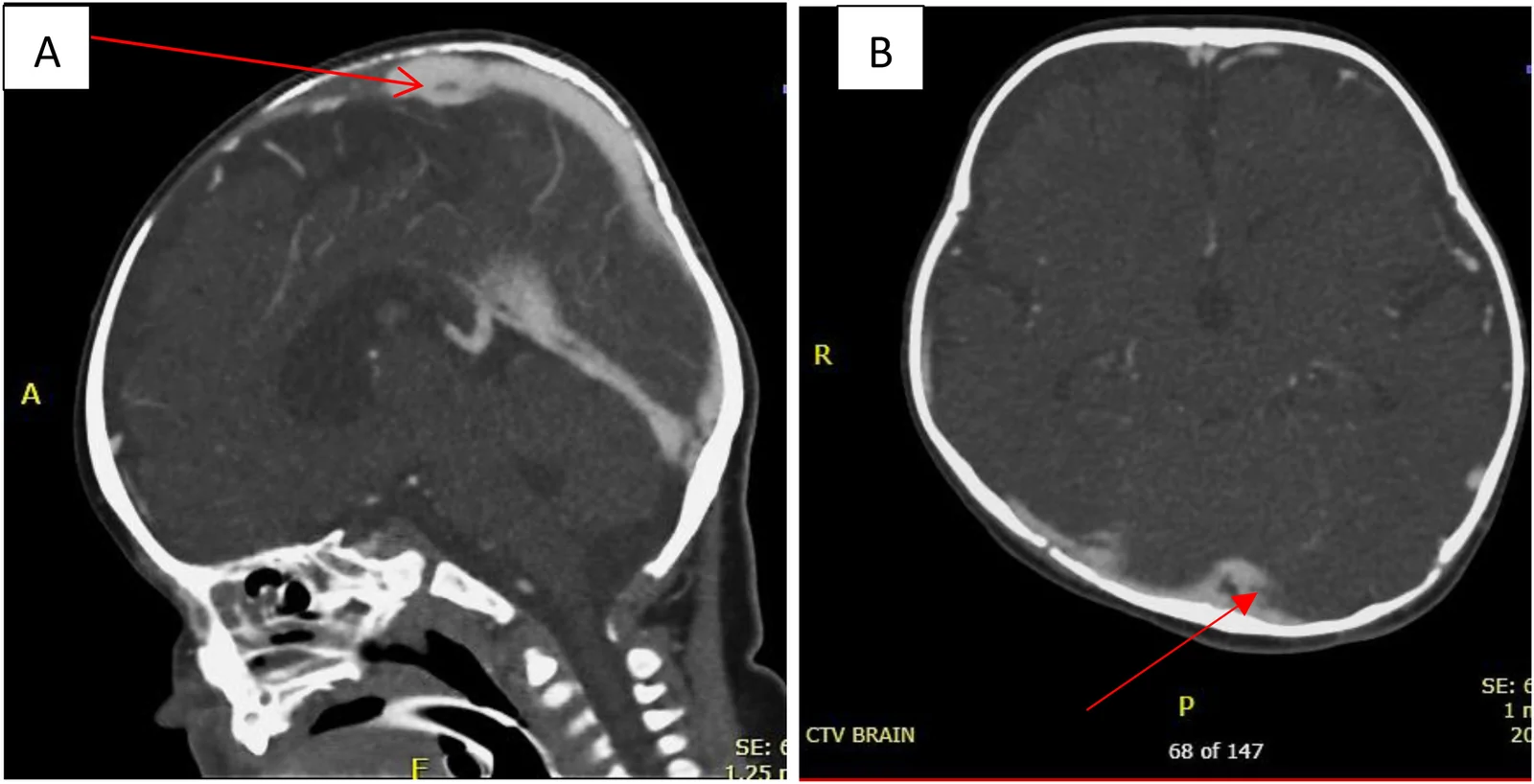
Brain computed tomography venogram (CTV) showing partial non-occlusive filling defect seen within the sinus confluence, straight sinus and left transvers sinus denoting sinus dural venous thrombosis. A; sagittal and B; axial plane.

Due to escalating respiratory distress and impending respiratory failure, the patient was intubated on the first day of admission. The neurology team was consulted regarding the reported seizure history. Continuous electroencephalogram monitoring revealed normal background activity. Levetiracetam was initiated with a loading dose followed by maintenance therapy; the neurological examination remained non-focal. Urgent computed tomography (CT) and CT venography (CTV) demonstrated non-occlusive CVST involving the confluence of sinuses, the straight sinus, and the left transverse sinus, with no evidence of intracranial hemorrhage (Figure 3). A lumbar puncture and cerebrospinal fluid analysis were performed, yielding normal results and negative cultures.

**Figure 4:**
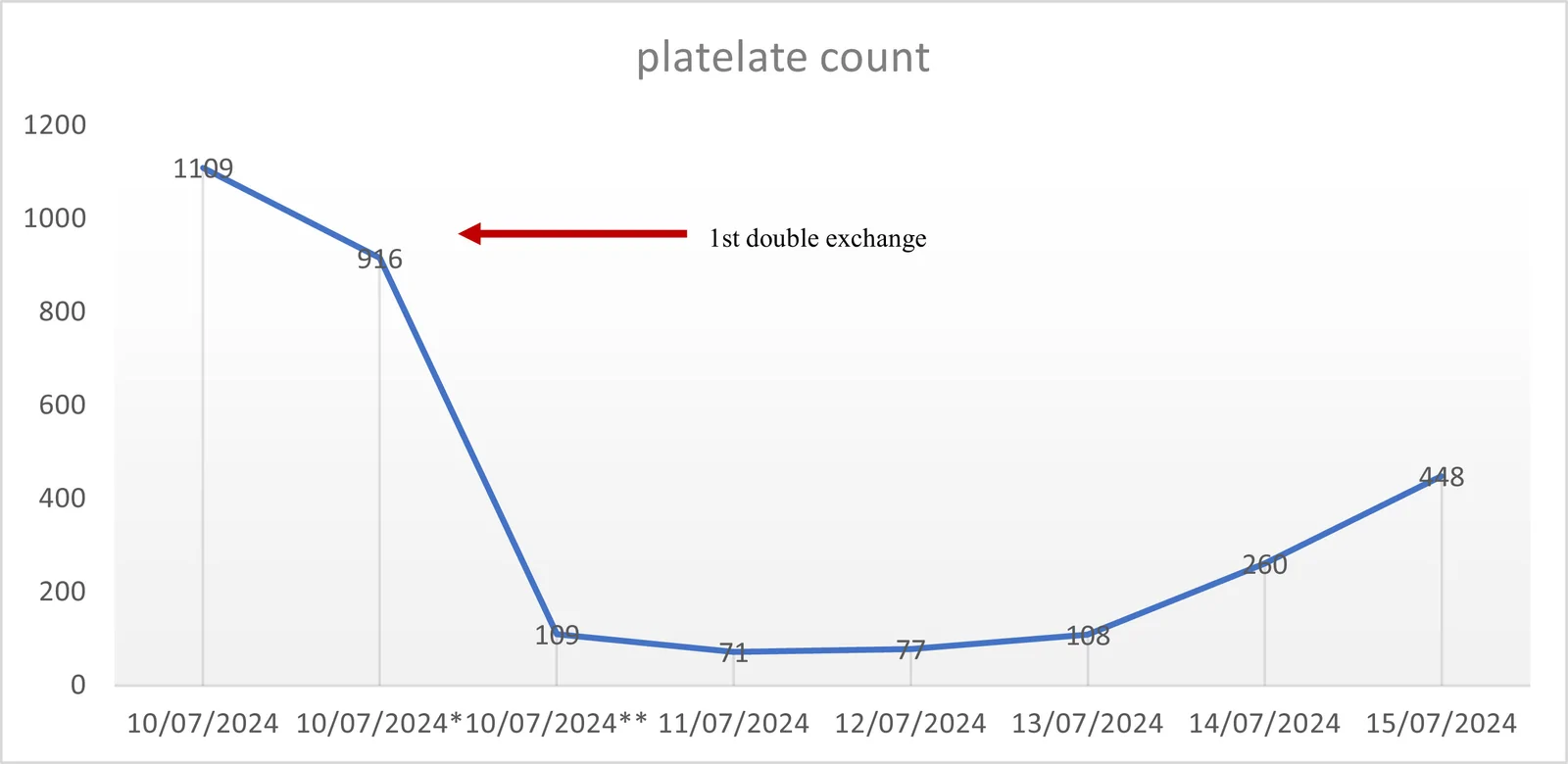
Platelet count by date during the pertussis course.

Given the severe hyperleukocytosis, thrombocytosis, respiratory failure, and CVST, an urgent double-volume exchange transfusion was performed over 2.5 h in two cycles without significant complications. A post-exchange complete blood count showed a marked reduction in WBC to 27.3 × 10⁹/L and platelets to 109 × 10⁹/L (Figures 1& 4). After multidisciplinary consultation between pediatric neurology and hematology, therapeutic unfractionated heparin was started with informed parental consent. Over the following 24 h, two additional single-volume exchange transfusions were required due to recurrent leukocytosis (WBC peaking at 52.4 × 10⁹/L and 41.6 × 10⁹/L). These subsequent procedures successfully reduced the WBC counts to a range of 17–19 × 10⁹/L and the platelet count to 77–109 × 10⁹/L (Figures 1& 4).

**Figure 5:**
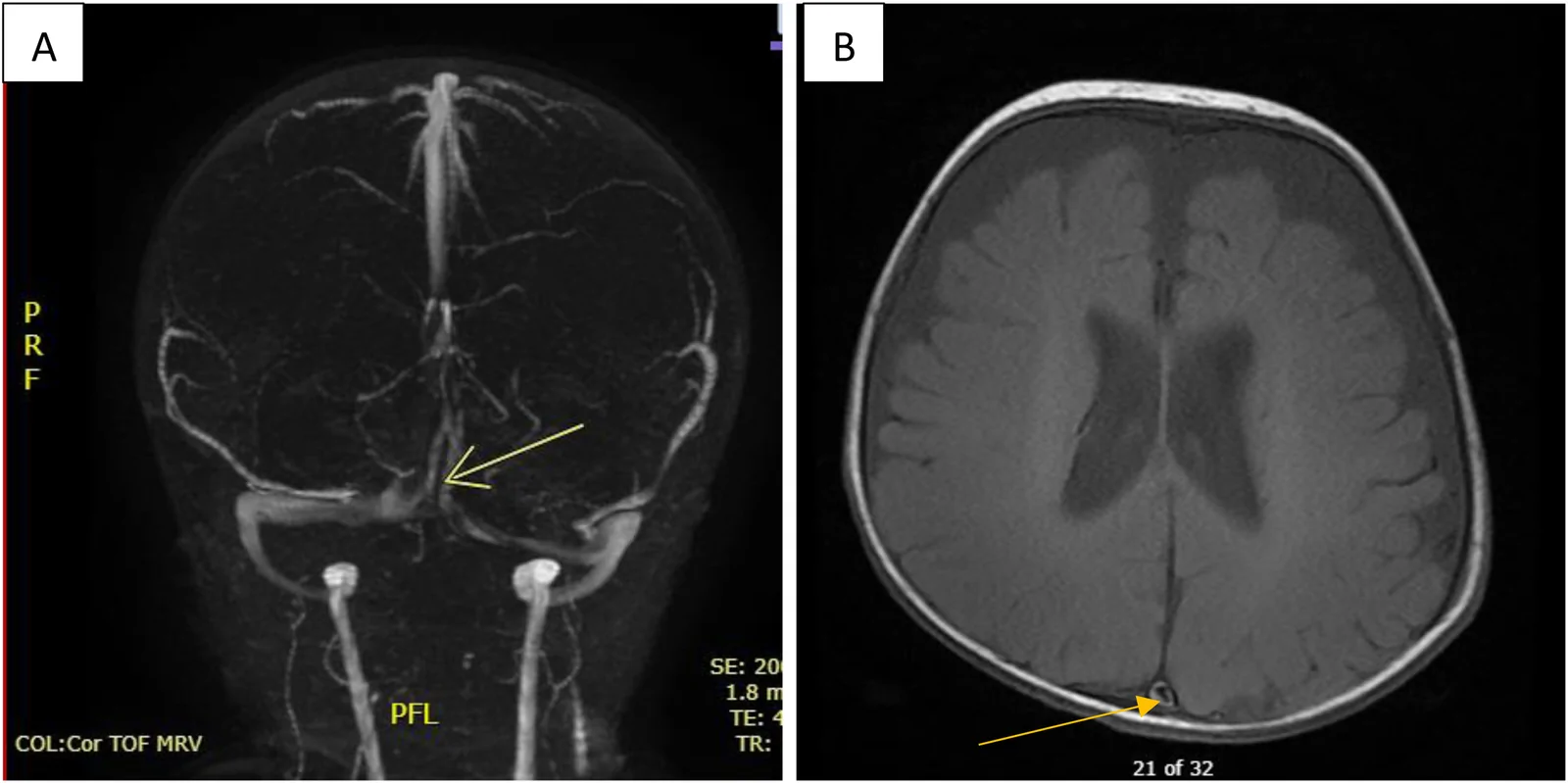
Brain magnetic resonant venogram (MRV) showing non-occlusive dural venous thrombosis of the sinus confluence and the left transvers sinus to involve superior sagittal sinus A; coronal and B; axial plane.

The patient was also treated with a course of intravenous cefepime and oral azithromycin. A follow-up brain MRI and MR venography performed one week after admission showed mild progression of the previously noted thrombosis, which showed extension of the thrombosis to the superior sagittal sinus (Figure 5). Therapeutic heparin was continued and later transitioned to subcutaneous enoxaparin after two weeks. Throughout hospitalization, the neurological examinations remained normal, with no additional abnormal seizures.

Echocardiography revealed normal pulmonary artery pressure. The patient was successfully extubated on Day 11 and transitioned to a high-flow nasal cannula, subsequently being weaned to low-flow oxygen. He remained clinically stable and was transferred to the general pediatric ward after 22 days. He was discharged home on Day 29 of hospitalization in good condition.

After a month, an outpatient follow-up with MRI and MRV showed interval improvement in the thrombosis involving the superior sagittal, straight, and left transverse sinuses, with residual peripheral non-occlusive filling defects. At subsequent follow-ups, the patient demonstrated normal growth and development, and neurological examinations remained within normal limits.

## Discussion

CVST is a rare but potentially life-threatening condition associated with high morbidity. To our knowledge, this is the first case to emphasize the association between *B. pertussis* and the development of CVST. We present a unique case of an unvaccinated infant with severe pertussis, hyperleukocytosis, and thrombocytosis who presented with seizures. This case highlights the necessity of maintaining a high index of suspicion for CVST in such patients. Early neuroimaging is critical to facilitate timely leukoreduction and prevent irreversible neurological damage.

Despite the widespread implementation of effective vaccination programs, outbreaks and endemic transmission of *B. pertussis* continue to occur globally [26]. Pertussis remains a significant public health concern, particularly in infants under one year of age, with cyclical epidemics reported every 2-5 years, even in countries with high vaccination coverage [27,28]. Among infectious diseases, pertussis is a leading cause of infant morbidity and mortality. The risk of severe outcomes is notably heightened when the infection is complicated by pulmonary or neurological involvement [24].

Although pertussis is primarily a respiratory illness, its systemic manifestations, particularly hematologic and neurologic, are increasingly being recognized, especially among unvaccinated infants. Severe neurological complications have been reported in 6%–34% of infants with pertussis, with the highest incidence observed in those younger than six months [29,30]. Karen et al. reported that infants under two months of age have the highest rates of pertussis-related hospitalization (82%), pneumonia (25%), seizures (4%), encephalopathy (1%), and mortality (1%) [23]. Given these findings, our under 2-month-old, unvaccinated patient was at significantly elevated risk for developing severe complications. Litvak et al. observed that CVST is one of the complications associated with pertussis, as identified in autopsies. Although this study has historical and methodological limitations, it provides evidence that CVST has been observed in fatal cases of whooping cough [24]. The lack of recent reports may reflect underdiagnosis, as neuroimaging is not routinely performed in infants with pertussis and seizures.

In a cohort study involving 144 patients with severe pertussis, Tingting Shi et al. reported that the most common complication was pneumonia (70.1%), followed by respiratory failure (24.3%), septic shock (10.4%), pleural effusion (9.7%), and pulmonary hypertension (9.0%) [31]. Although less frequent, encephalitis and encephalopathy remain serious complications; U.S. surveillance data estimate the incidence of encephalopathy at 1.4%, with the highest rates observed in infants under two months of age [23]. Menkes and Halperin et al. documented seizures in 1.9%–2.3% of pertussis cases, and encephalopathy in 0.3%–0.5% [32,33]. These neurological complications are believed to result from a combination of bacterial toxin-mediated injury, asphyxia, hypercapnia, and cerebral vascular dysregulation. Notably, such events typically occur during the paroxysmal phase of illness, corresponding to peak bacterial lysis and endotoxin release [24,32]. In the present case, the seizure occurred during the second week of illness, aligning with this pathophysiological timeline.

M.J. Peters hypothesized that extreme leukocytosis in pertussis may lead to lymphocyte aggregation within the pulmonary vasculature, increasing vascular resistance through mechanical obstruction rather than hypoxic vasoconstriction [34]. Although this mechanism has been primarily studied in the context of pulmonary hypertension, it may extend to the cerebral venous system. Such a process could provide a potential, but unproven pathophysiological basis for the development of CVST in our patient.

Several mechanisms may link pertussis to CVST. First, hyperleukocytosis increases blood viscosity and promotes venous stasis, both of which are key components of Virchow's triad. Second, toxins produced by *B. pertussis* may cause endothelial dysfunction, thereby raising the risk of thrombosis. Third, hypoxia and dehydration resulting from persistent coughing and apnea can worsen a hypercoagulable state. Lastly, systemic inflammation can activate coagulation pathways [34]. Our patient presented during the paroxysmal stage of pertussis, characterized by the production of toxins that are the primary cause of pathology at this stage. At this stage, leukocytosis peaked, significantly increasing the likelihood of venous stasis. Coupled with respiratory symptoms and persistent coughing fits (whooping), this predisposed him to brain hypoxia.

Although pulmonary hypertension was not observed in this patient, as confirmed by the echocardiographic evaluation, he developed significant respiratory distress and seizures in the context of a rapidly escalating WBC count, rising from 15 × 10^9^/L to 52 × 10^9^/L, then to 93 × 10^9^/L despite aggressive hydration. This clinical course aligns with existing literature that associates severe hyperleukocytosis in pertussis with both respiratory and neurological complications, even in the absence of overt pulmonary hypertension [35].

Previous studies have indicated that platelet counts tend to be elevated during inflammation and various infections. Numerous investigations have established a connection between thrombocytosis and pertussis, especially during the paroxysmal phase of the illness [20,36,37]. In the current case, the platelet count during this stage was extremely high, exceeding 1000 × 10⁹/L. This elevation may have contributed to the development of CVST. Additionally, thrombophilia is a recognized risk factor for CVST, which could have potentially explained this patient’s condition. However, the negative result from his thrombophilia test ruled out this possibility. Collectively, these factors explain the increased risk for the development of CVST and, consequently, convulsions.

In this case, the diagnosis of dural sinus thrombosis was confirmed by neuroimaging, prompting initial treatment with unfractionated heparin in combination with blood exchange transfusion for leukoreduction, followed by a three-month course of therapeutic subcutaneous low-molecular-weight heparin (LMWH). The management of CVST in infants with pertussis presents unique clinical challenges. One primary consideration is balancing the use of anticoagulation therapy with the risk of bleeding, particularly in the presence of hemorrhagic infarctions and thrombocytopenia post-exchange transfusion, necessitating close monitoring of neurological and coagulation status. Although the evidence base for CVST treatment in neonates and infants continues to evolve, current guidelines recommend early initiation of anticoagulation with LMWH, unless contraindicated [38]. Given the high risk of bleeding in this patient and after obtaining parental consent, the multidisciplinary team decided to start unfractionated heparin, which has a short half-life with an antidote unlike LMWH. Then the guidelines recommendation of therapeutic LMWH after two weeks was followed. Follow-up MRI and MR venography demonstrated successful regression of the thrombosis. The patient was subsequently monitored in the outpatient setting by the neurology team, with serial neurological examinations remaining normal and no further seizures reported.

The mainstay of treatment for pertussis is optimal supportive care, including respiratory support, leukoreduction in severe hyperleukocytosis cases, and infection control, all of which are essential to improve patient outcomes. While exchange transfusion has been the primary modality reported to improve survival in patients with critical pertussis, it has the potential of dual effects [11,39]. First, leukoreduction is expected to reduce WBC aggregation in the small pulmonary vessels [4]. Second, the resultant exchange transfusion may reduce circulating pertussis toxins, which also contribute by inhibiting G proteins, leading to cardiopulmonary dysfunction [8]

Management in this case successfully reduced the leukocyte count through double volume exchange transfusions. When the patient's WBC count peaked at 93.3 × 10^9^/L, the procedure was performed without significant complications, effectively lowering the count to 27.3 × 10^9^/L. Subsequently, single-volume exchange transfusions were performed on two occasions when the WBC count rebounded to 52.4 × 10^9^/L and 41.6 × 10^9^/L, respectively (Figure 4). This modality of management of life-threatening pertussis complicated by hyperleukocytosis and pulmonary hypertension using leukoreduction by exchange transfusion or leukapheresis can effectively prevent complications as reported in previous studies [40,41]. Additionally, Rowlands et al. found that the addition of leukodepletion by exchange transfusion in the intensive care of infants with critical pertussis reduced the mortality rate from 44%−10% [42]. Although we initially attempted to reduce the WBC count through hyperhydration, this approach did not yield significant clinical benefits in our patient. This experience contrasts with the findings of Lashkari et al., who reported hyperhydration as an effective adjunct treatment for leukocytosis in their cohort [43]. In a study of ten infants with critical pertussis who received exchange transfusion, Nieves et al. concluded that the treatment may be ineffective if organ failure has already occurred [44]. Early implementation of leukoreduction therapy may significantly improve clinical outcomes. Furthermore, previous authors have noted that even when hyperleukocytosis is not the immediate cause of mortality, exchange transfusion remains beneficial by reducing circulating pertussis toxin levels, thereby mitigating its systemic, life-threatening effects. The current patient was extubated a week after the procedure in a stable clinical condition.

Although no studies have shown a direct link between CVST and pertussis due to the absence of routine neuroimaging in these cases, there is also no evidence to suggest a correlation between CVST in pertussis and thrombocytosis. However, it is worth considering whether early intervention targeting thrombocytosis could prevent such devastating neurological complications during the course of pertussis.

However, a more recent clinical trial of pertussis intravenous immune globulin (P-IGIV) treatment found no significant benefit in disease symptoms, although leukocytosis was not measured in this study [45]. Very recently, Nguyen et al. administered humanized murine monoclonal antibodies specific for pertussis toxin to experimentally *B. pertussis*-infected animals [46]. They found that this treatment was more effective than P-IGIV in preventing leukocytosis in mice and was effective in reducing leukocytosis when administered therapeutically to infected baboons. In the future, this may lead to a more specific and compelling antibody-based therapy for treating leukocytosis and other pertussis toxin-associated effects in humans. P-IGIV is not available at our center, and the leukocyte count was successfully reduced without rebound by exchange transfusion.

This appears to be a rare event, perhaps because it has not been previously identified or simply because it remains underreported despite the high incidence of pertussis and its frequent presentation with extreme leukocytosis in infants under two months of age. While it is well-established that many infants in this demographic develop severe pertussis with marked leukocytosis, the scarcity of literature suggests that hyperleukocytosis may not be the sole cause of CVST in this patient

This report has several limitations. First, thrombophilia screening was not performed, and information about family history of thrombophilia was not obtained which limit the ability to rule out an inherited predisposition. Second, although platelet trends were recorded, platelet function testing and coagulation factor assays are unavailable. Third, as a single case report, causality between hyperleukocytosis and CVST cannot be established.

## Conclusion

This case emphasizes the rare but serious possibility of CVST as a neurological complication in infants suffering from severe pertussis, especially in those who exhibit significant hyperleukocytosis and thrombocytosis. While leukocytosis and leukostasis may lead to venous stasis, other contributing factors, such as platelet activation, endothelial injury, and thrombophilia, likely play a role in thrombogenesis, potentially activated by pertussis toxins. Clinicians should maintain a high level of suspicion for CVST in infants with pertussis who present with seizures, altered consciousness, or focal neurological signs. Early neuroimaging and prompt management of hematologic abnormalities, including leukoreduction and judicious anticoagulant use, may improve neurological outcomes. However, further research is necessary to understand better the relationship between leukocytosis, thrombocytosis, and toxin-mediated vascular injury, as well as to determine the threshold for CVST in cases of pertussis. This will help develop more effective treatment strategies and identify optimal leukoreduction thresholds for managing pertussis.

## Conflicts of interest

The authors declare no conflicts of interest.
